# Variability of temperature measurements recorded by a wearable device by biological sex

**DOI:** 10.1186/s13293-023-00558-z

**Published:** 2023-11-01

**Authors:** Lauryn Keeler Bruce, Patrick Kasl, Severine Soltani, Varun K. Viswanath, Wendy Hartogensis, Stephan Dilchert, Frederick M. Hecht, Anoushka Chowdhary, Claudine Anglo, Leena Pandya, Subhasis Dasgupta, Ilkay Altintas, Amarnath Gupta, Ashley E. Mason, Benjamin L. Smarr

**Affiliations:** 1 UC San Diego Health Department of Biomedical Informatics, University of California San Diego, San Diego, CA USA; 2 Shu Chien-Gene Lay Department of Bioengineering, University of California San Diego, 9500 Gilman Dr, , La Jolla, San Diego, CA USA; 3 Bioinformatics and Systems Biology, University of California San Diego, San Diego, CA USA; 4 Department of Electrical and Computer Engineering, University of California San Diego, La Jolla, CA USA; 5 Osher Center for Integrative Health, University of California San Francisco, San Francisco, CA USA; 6 Department of Management, Zicklin School of Business, Baruch College, The City University of New York, New York, NY USA; 7 Halıcıoğlu Data Science Institute, University of California San Diego, San Diego, CA USA; 8 San Diego Supercomputer Center, University of California San Diego, San Diego, CA USA

## Abstract

**Background:**

Females have been historically excluded from biomedical research due in part to the documented presumption that results with male subjects will generalize effectively to females. This has been justified in part by the assumption that ovarian rhythms will increase the overall variance of pooled random samples. But not all variance in samples is random. Human biometrics are continuously changing in response to stimuli and biological rhythms; single measurements taken sporadically do not easily support exploration of variance across time scales. Recently we reported that in mice, core body temperature measured longitudinally shows higher variance in males than cycling females, both within and across individuals at multiple time scales.

**Methods:**

Here, we explore longitudinal human distal body temperature, measured by a wearable sensor device (Oura Ring), for 6 months in females and males ranging in age from 20 to 79 years. In this study, we did not limit the comparisons to female versus male, but instead we developed a method for categorizing individuals as cyclic or acyclic depending on the presence of a roughly monthly pattern to their nightly temperature. We then compared structure and variance across time scales using multiple standard instruments.

**Results:**

Sex differences exist as expected, but across multiple statistical comparisons and timescales, there was no one group that consistently exceeded the others in variance. When variability was assessed across time, females, whether or not their temperature contained monthly cycles, did not significantly differ from males both on daily and monthly time scales.

**Conclusions:**

These findings contradict the viewpoint that human females are too variable across menstrual cycles to include in biomedical research. Longitudinal temperature of females does not accumulate greater measurement error over time than do males and the majority of unexplained variance is within sex category, not between them.

## Abstract (plain language summary)

Women are still excluded from research disproportionately, due in part to documented concerns that menstrual cycles make them more variable and so harder to study. In the past, we have challenged this claim, finding it does not hold for animal physiology, animal behavior, or human behavior. Here we are able to show that it does not hold in human physiology either. We analyzed 6 months of continuously collected temperature data measured by a commercial wearable device, in order to determine if it is true that females are more variable or less predictable than males. We found that temperatures mostly vary as a function of time of day and whether the individual was awake or asleep. Additionally, for some females, nightly maximum temperature contained a cyclical pattern with a period of around 28 days, consistent with menstrual cycles. The variability was different between cycling females, not cycling females, and males, but only cycling female temperature contained a monthly structure, making their changes more predictable than those of non-cycling females and males. We found the majority of unexplained variance to be within each sex/cycling category, not between them. All groups had indistinguishable measurement errors across time. This analysis of temperature suggests data-driven characteristics might be more helpful distinguishing individuals than historical categories such as binary sex. The work also supports the inclusion of females as subjects within biological research, as this inclusion does not weaken statistical comparisons, but does allow more equitable coverage of research results in the world.

## Highlights

- Sex does not separate more and less variable individuals, as measured in continuous body temperature.
- Analysis of high-resolution, longitudinal temperature detailed here supports the inclusion of females in biomedical research.
- Cycling female variance in temperature is more structured across days and months than males, but most variance is within groups, and not between them.
- This work includes methods for identifying cycles in participant data without pre-existing labels.

## Background

Females represent roughly half of humanity, and as such, are worth equal consideration in health research. Nevertheless, there persists an underrepresentation of females in both animal research [1–4] and human clinical trials [5–8]. As researchers themselves reported in anonymous surveys, this resistance to using female subjects, despite policies of inclusion, partially stems from the assumption that including female subjects will increase the heterogeneity of study results by virtue of having ovarian rhythms (estrus or menses, respectively) [4]. Coupled with the assumption that results from males will generalize to females, this lack of inclusion leads to serious inequalities in female health outcomes and available treatments (e.g., [9–11]).

The assumption that females are more variable than males has been explored in mice and rats [1, 4, 12]. Variability has not been found to be significantly greater in females than males for most traits. On the contrary, for many traits, variability was substantially greater in males over a range of behavioral, neurobiological, and physiological traits [12–15]. Continuous activity and core body temperature (CBT) in mice also revealed higher intra- and inter-individual variability in male mice than in females by a range of statistical comparisons [16, 17]. Quantitative comparisons in rodent models refute the common assumption of greater variability in female subjects. Analogous efforts in humans using online activity data as a proxy for biological rhythms have aligned with the animal literature, finding small differences that evince males as marginally more variable than females across timescales [18].

Human biometrics are not static, but continuously change in response to stimuli, and/or as part of dynamic equilibria driven by variable-cycle feedback loops [19, 20]. These changes occur across timescales, including daily rhythms and sometimes longer hormonal rhythms, as in menses. The exact dynamics are driven by the experience of the individual, and as such differ from person to person. While actigraphy is commonly used for longitudinal assessment in animal models and in humans, underlying physiological dynamics may continue oscillating while activity remains at 0 during inactivity; activity rhythms may also be masked by behavioral and social impositions (i.e., school or work schedules, etc*.*). We have found body temperature to be a superior modality of continuous monitoring, with ties to hormonal changes, daily rhythms, and women’s health states [21–25].

Body temperature changes over time, as well as with gender, age, environment, stage of menstruation, etc. [24]. Mean sex differences in body temperature are documented, but population means do not describe variability over multiple timescales or across individuals comprising a population [26, 27]. To date, studies have not been carried out using longitudinal physiology data to assess how these variables change within individual humans over time, and how such changes impact the results of statistical comparisons.

Here, we use data from an off-the-shelf wearable device (Oura Ring, Oura Health Oy, Oulu, Finland) gathered under the umbrella of the TemPredict study to develop COVID-19 detection capabilities [28]. We analyze 300 females and 300 age-matched males from this data set. We use continuous temperature to generate representative statistical measures of inter- and intra-individual variability across multiple timescales to assess the extent to which female sex correlates to increased variability in all of these instances, and the extent to which differences between sexes impede statistical comparisons in one group relative to another.

## Results

**Fig. 1:**
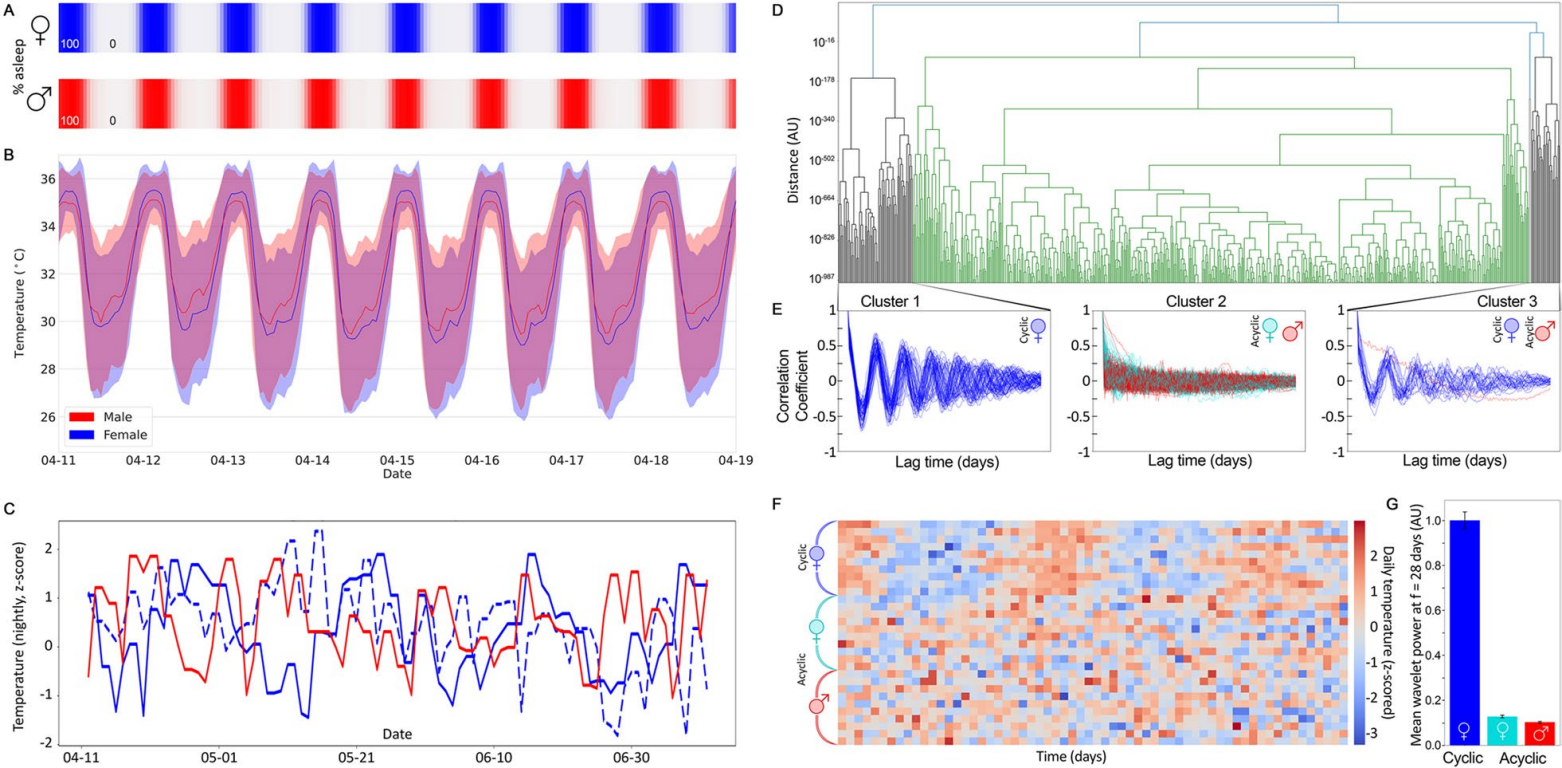
**A** Heatmap of % of male (red, top) and female (blue, bottom) individuals in sleep per hour during the example week of April 11, 2020 to April 18, 2020. **B** Mean ± standard deviation of hourly temperature deviation over 300 females (blue) and 300 males (red) covering a single week (mean line ± standard deviation fill). **C** Temperature maximums for two females (blue apparently cyclic: solid; apparently acyclic: dashed) and one male (red) over 3 months. **D** Hierarchical clustering performed to classify individuals as cyclic or acyclic using dynamic time warping distance of autocorrelation of nightly temperature maximum. **E** Autocorrelation profiles for individuals within each cluster resulted in classification of individuals as cyclic, acyclic, and cyclic for clusters 1, 2, and 3, respectively. **F** Heatmap of 3 months of nightly maximum temperature data 10 cyclic female (chosen from the 300 for their rough cycle alignment to illustrate the cyclicity), acyclic female, and acyclic males. **G** Mean and standard error of the 26- to 32-day power band from the wavelet power spectra generated from temperature max data for each category (cyclic females: blue; acyclic females: teal; males: red)

Average and standard deviation of hourly temperature confirmed daily variation in temperature by both sexes when asleep or awake (Fig. 1A, B). A monthly pattern of variation in temperature was also present in some females (Fig. 1C, solid blue line), indicating ovulatory dependency of temperature as expected, and allowing for classification of individuals as cyclic or acyclic. Through hierarchical clustering of autocorrelation profiles (Fig. 1D), we found three clusters (Fig. 1E), with all cyclic female individuals (*n* = 72) in the first cluster, all acyclic individuals (female *n* = 195; male *n* = 299) in the second cluster, and both female cyclic individuals with lower amplitude autocorrelation values (*n* = 33) and a single acyclic male in the third. The single male in the third cluster was assigned as acyclic after visual inspection, as no 28-day oscillation of temperature was present, despite their distance value determined by dynamic time warping having automatically placed them in cluster three. Based on this clustering, individuals were assigned to one of three categories: cyclic females (all females from clusters 1 and 3), acyclic females (all females from cluster 2), and acyclic males (all males). Ovarian cycle-like 20–30 day oscillations of nightly temperature [29] were consistently detected in cyclic females but acyclic females and males lacked such periodicity (Fig. 1F, G).

**Fig. 2:**
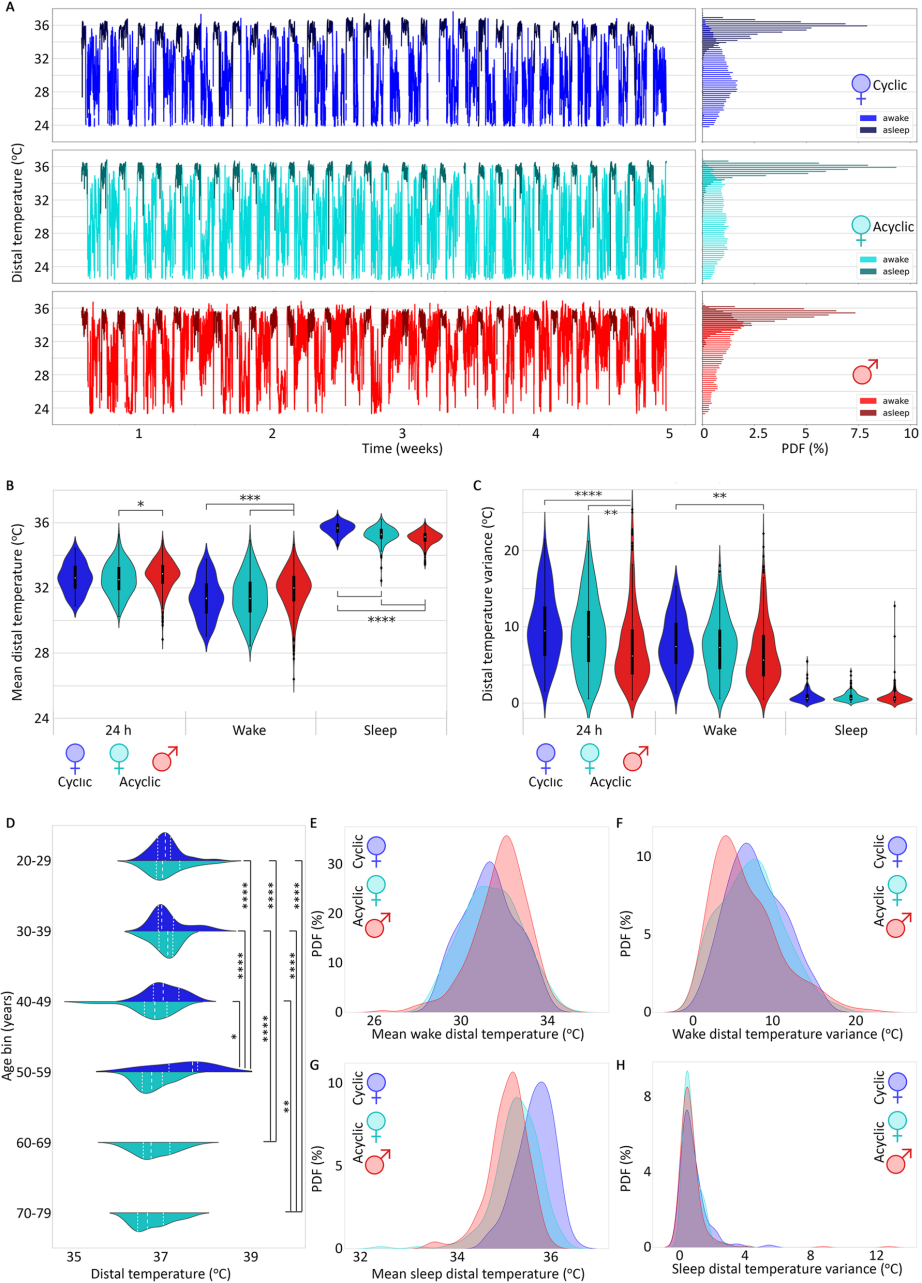
Daily temperature profiles (**A**) for a representative individual from the cyclic female (blue), acyclic female (teal), and acyclic male (red) categories. Temperatures when asleep (darker shades) show higher distal body temperature with a smaller range than when awake. Violin plot of mean temperature by category (**B**) and variance (**C**) across all 24 h (left), only wake times (center), and only sleep times (right). Violin plot of nightly maximum temperature (**D**) for females subset by age bin, split by cyclic status, with quantiles in white. Kernel density estimate for average temperature by category during wake (**E**) and sleep (**G**). Kernel density estimate for temperature variance by category during wake (**F**) and sleep (**H**). Bonferroni corrected *p*-value annotations for 9 comparisons: *: 1.00e−3 < *p* <  = 6.00e−3, **: 1.00e−4 < *p*, ***: 1.00e−5 < *p*, ****: *p* <  = 1.00e−5

Minute-level data for a single month allowed for visual comparison of temperature variance across timescales (Fig. 2A, left). All individuals showed a wider variance in temperature while awake, but the distribution of awake temperature values was distributed fairly evenly for the acyclic female, skewed lower for cyclic females, and skewed higher for males (Fig. 2A, right). We found differences in mean temperature for each sex/cyclicity group at three time scales (24 h, when asleep, and when awake; Fig. 2B). Males had significantly higher mean temperature than acyclic females for 24 h temperature (*p* = 0.003, *U* = 2.5e4); males had significantly higher wake temperatures than both cyclic and acyclic females (cyclic vs. male *p* = 6.5e−5, acyclic female vs. male *p* = 8.6e−5, *U* = 2.3e4); cyclic females had significantly higher sleep temperatures than acyclic females, who were significantly higher than males (cyclic vs. male *p* = 5.6e−27, *U* = 2.6e4; acyclic female vs. male *p* = 3.2e−7, *U* = 3.7e4; cyclic vs. acyclic female *p* = 2.5e−12, *U* = 1.5e4; Fig. 2D, Table 1). Variance across 24 h was significantly lower in males than in cyclic or acyclic females (*p* = 1.1e−7, *U* = 2.1e4 and *p* = 1.6e−4, *U* = 3.5e4, respectively). Only cyclic females had significantly higher wake temperature variance than males (*p* = 3.1e−4, *U* = 1.9e4, Fig. 2C). Analysis of means and standard deviations of distal body temperature for females and males, separated into six age bins, revealed no significant differences between age bins for males (data not shown). By contrast, in the female subset, significant differences were only seen between bin pairs where one was below 50 years old and the other above 50 years old (statistics in Table 1; Fig. 2D).

**Table 1:** Bonferroni corrected Mann–Whitney–Wilcoxon two-sided test *p*-values (*U* test statistics) of comparisons of median nightly maximum temperature between females in age groups below and above 50 years old

Age Bins	50–59	60–69	70–79
20–29	5.7e−8**** (2e3)	5.5e−6**** (1.9e3)	1.7e−8**** (2.1e3)
30–39	5.7e−8**** (2e3)	2.7e−6**** (1.9e3)	2.2e−8**** (2.1e3)
40–49	1.9e−3* (1.7e3)	0.01 (1.62e3)	5.7e−4** (1.8e3)

Bonferroni corrected *p*-value annotations for 15 comparisons: *: 6.7e−4 < *p* <  = 3.00e−3, **: 6.7e−5 < *p*, ***: 6.7e−6 < *p*, ****: *p* <  = 6.7e−6

The kernel density estimates of temperature mean for each category (Fig. 2E–G) showed large overlaps between categories. For awake mean temperature, Cohen d’s effect sizes were small to medium for each pairwise comparison: cyclic female vs. acyclic female = − 0.07, cyclic female vs acyclic male = − 0.41, and acyclic female vs. acyclic male = − 0.33. Effect sizes for comparing awake mean temperatures were the largest between cyclic females and acyclic males: cyclic female vs. acyclic female = 0.85, cyclic female vs acyclic male = 1.40, and acyclic female vs. acyclic male = 0.41. Categorical distributions of the variance (Fig. 2F and H) also substantially overlapped, with lower overall effect sizes in each pairwise comparison (awake temperature variance: cyclic female vs. acyclic female = 0.16, cyclic female vs acyclic male = 0.31, and acyclic female vs. acyclic male = 0.17; asleep temperature variance: cyclic female vs. acyclic female = 0.08, cyclic female vs acyclic male = 0.12, and acyclic female vs. acyclic male = 0.06).

**Fig. 3:**
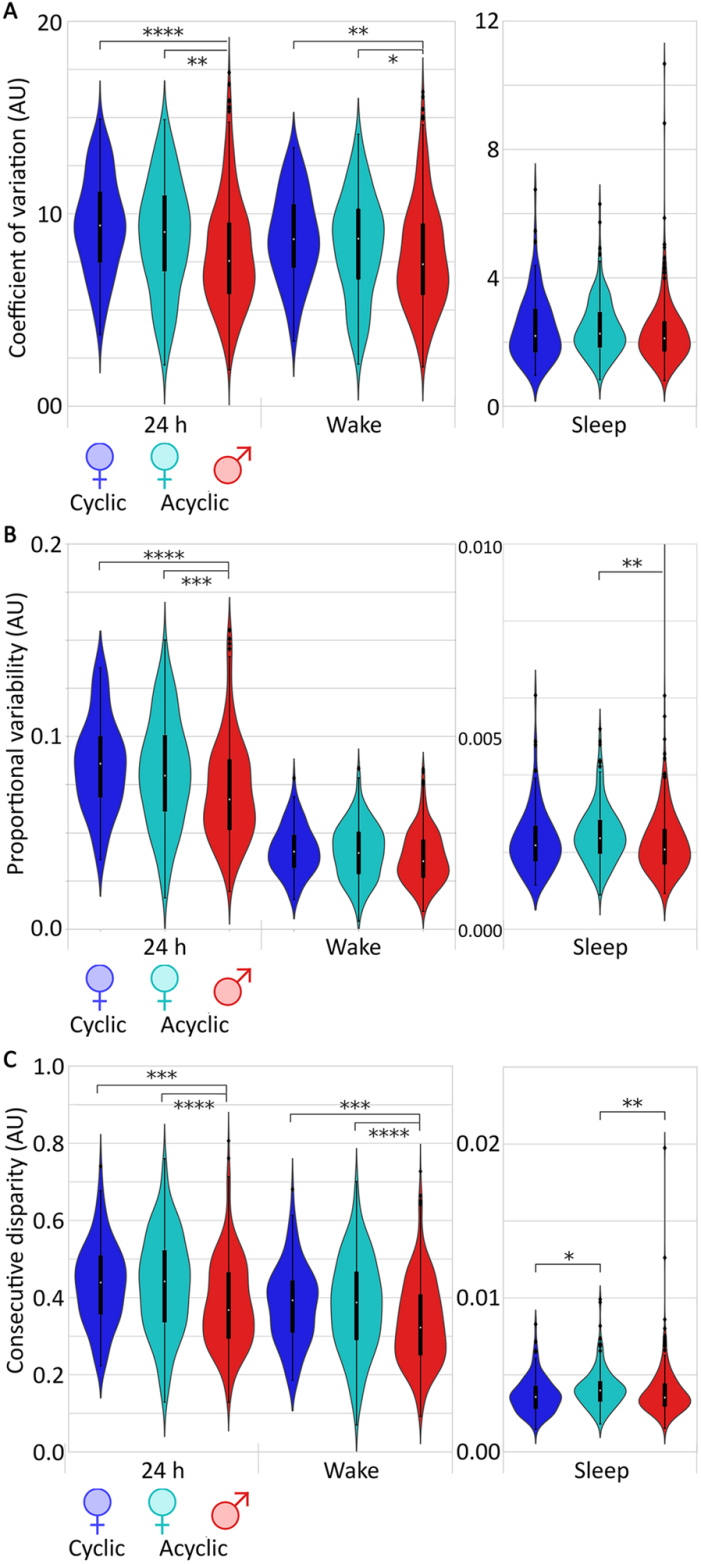
Violin plot of average **A** coefficient of variation (CV), **B** proportional variability index (PV), and **C** consecutive disparity index (D)﻿ for cyclic females (blue), acyclic females (teal), and males (red) for timepoints for all 24 h, only when awake, or only when asleep. Bonferroni corrected *p*-value annotations for 9 comparisons: *: 1.0e-3 < *p* <  = 6.00e−3, **: 1.0e−4 < *p*, ***: 1.0e−5 < *p*, ****: *p* <  = 1.0e−5

Three submetrics of daily variability were assessed per individual per category. Coefficient of variation (CV) showed significant differences between female and male categories for the 24 h and awake time frames, with cyclic and acyclic females showing higher mean levels of dispersion, while the male population showed greater interindividual variability of CV (24 h: cyclic female vs male *p* = 3.8e−7, *U* = 2.1e4; acyclic female vs male *p* = 1.5e−4, *U* = 3.5e4. Wake: cyclic female vs male *p* = 1.8e−4, *U* = 2e4; acyclic female vs male *p* = 5.4e−3, *U* = 3.4e4; Figure 3A). The proportional variability (PV) index, showed similar results, with the mean PV of cyclic females only higher than males at the 24-h timescale (24 h: cyclic female vs male *p* = 3e−7, *U* = 2.1e4; acyclic female vs male *p* = 1.9e−5, *U* = 3.6e4. sleep: acyclic female vs male *p* = 2.5e−4, *U* = 3.5e4 Fig. 3B). A variable sensitive to temporal autocorrelation, the consecutive disparity index (D) was low for all populations, and was significantly different between the female and male groups for the 24 h and awake time points (24 h: cyclic female vs male *p* = 2.4e−5, *U* = 2e4; acyclic female vs male *p* = 2.4e−6, *U* = 3.7e4. Wake: cyclic female vs male *p* = 5.0e−5, *U* = 2e4; acyclic female vs male *p* = 9.7e−6, *U* = 3.6e4) but during sleep, cyclic females were not significantly different from the male group and instead were different from acyclic females (cyclic vs acyclic female *p* = 0.002, *U* = 8.1e3; acyclic female vs male *p* = 8.8e−4, *U* = 3.4e4; Fig. 3C).

**Fig. 4:**
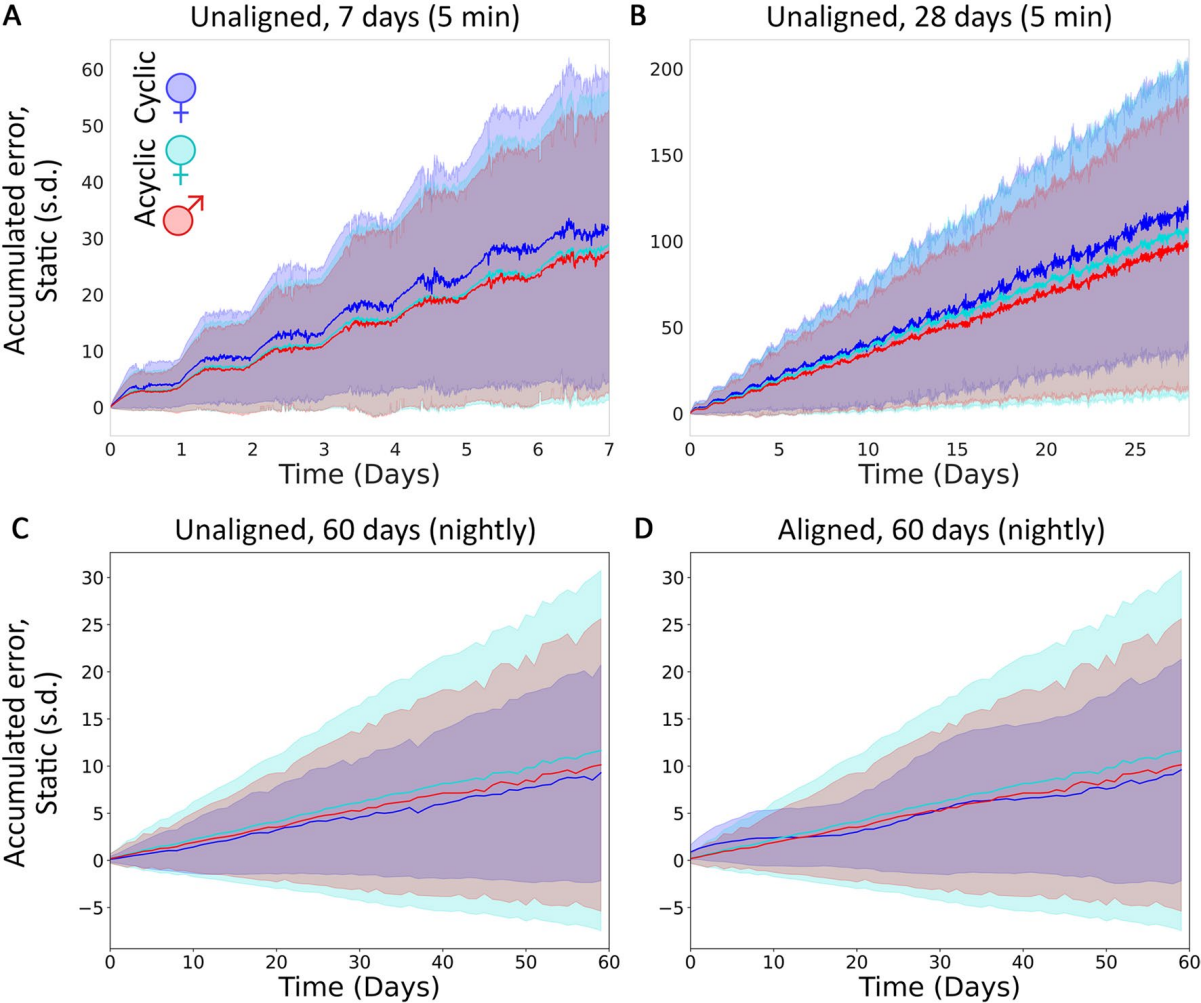
Cumulative error of 5-min resolution data for cyclic females (blue), acyclic females (teal), and males (red) when compared to a single static mean for 7 days (**A**) and 28 days (**B**) and 60 days (**C**) with cycling females either unaligned (natural cyclic phase distribution; **﻿C**) or aligned by cyclic phase (**D**)

Quantification of accumulated distance from population mean (termed “cumulative error” [16]) for each population (cyclic female, acyclic female, and male) revealed no significant differences between categories across 5-min resolution data for 7 and 28 days (Fig. 4, Bonferroni corrected *p*-value significance threshold is set to 0.0025 to account for the 4 comparisons, Fig. 4A: *p*-value: 0.43, *U* = 1.7; Fig. 4B: *p*-value = 0.03, *U* = 7.1). Cumulative error taken at nightly resolution across 2 months also resulted in no significant differences between categories (Fig. 4C: *p*-value: 0.57, *U* = 1.13; Fig. 4D: *p*-value = 0.43, *U* = 1.68). This held whether females were aligned by real world date (and so non-aligned by time of cycle) or by phase of the menstrual cycle (Fig. 4C, D). In the latter case, the error can be seen to take on a roughly 28 day wave pattern (Fig. 4D, blue shaded region), but with or without alignment by cycle, cyclic females show the least interindividual variance of cumulative error across months. In no case did cyclicity result in significant differences in cumulative error of distal body temperature from acyclic females or males when compared against a static population mean.

## Discussion

Analysis of variability in high resolution, continuous and longitudinal body temperature from large populations do not support the exclusion of females, cyclic or otherwise, from statistical analyses. Female participants did show significantly higher variability at daily timescales—a result of greater differentiation between sleep and wake—but in no case was this associated with a substantial effect size. Furthermore, this higher variance may in fact result from greater stability; wider night–day variance could be understood to reflect greater daily structure in these females. By comparison, males showed more overlap between night and day, which suggests less overall variance in temperature can be accounted for by time of day or sleep state in males. Consistent with this interpretation, males and females showed comparable amounts of cumulative error across timescales, despite the presence of menstrual cycles in some females. This work then continues to add similar findings to the literature of animal analogs, in which—despite ovulatory cyclicity—males are generally as variable as females. Whether this means male variance is truly less structured (more random) in time—or whether in other modalities beyond temperature the reverse is true—is likely dependent on the modality measured, and the species or population. Given the importance to future efforts at building time series-based algorithms for health, this question is worthy of deeper investigation in humans.

The numerical value of female temperature over time is highly dependent on whether or not their temperature is cycling, due to menstruation but possibly also due to other factors such as birth control methods. This analysis confirms that ovarian rhythms do affect temperature. This analysis does not suggest that these rhythms make any given measurement more prone to error. Even when comparing dynamic temperatures to a single, static mean for the population, error accumulation in all groups had no significant differences.

The analyses presented here suggest that there is substantial work still to do to develop reliable methods of characterizing variance over time in different human populations. It is worth noting, for example, that when categories showed differences, they were not always as might stereotypically be expected (e.g., temperature in cyclic females > acyclic females > males; or acyclic females = acyclic males). Furthermore, in all comparisons, the variance within each category vastly exceeds the differences between categories. This is consistent with a view in which traditional demographics (here, binary sex and decade of life) fail to account for the majority of the physiological variability, which appears to be within each category, rather than across categories. In the meantime, categories are still a useful construct, but many categories would benefit from longitudinal characterization. For example, we observed a difference between younger and older cycling females. This is presumed to be due to menopausal transitions, but as of this writing, there is no comparably high-resolution data-driven definition of perimenopause, nor description of how physiological changes (hot flashes, sleep disruption, chronotype, etc*.*) emerge in such data that could support separating individuals by “types” of perimenopause-related physiological patterns. Such descriptions would make topics like perimenopause, pregnancy, and menarche accessible to modern data modeling and precision predictive approaches.

We developed a method for labeling female participants as cyclic or acyclic using hierarchical clustering on the pairwise distance of time series temperature autocorrelation values. This tool is usable in data without participant-generated labels, which may be the case in many retrospective data analyses. Despite this, aligning multiple females by time of cycle still remains a challenge, as menstrual cycles vary in length and can be shifted due to environmental and hormonal factors, pregnancy, and birth control methods. We encourage future studies to gather additional information such as birth control methods (hormonal, IUD, etc.) and other reproductive conditions such as polycystic ovarian syndrome, pregnancy, and pregnancy complications such as preeclampsia.

### Perspectives and significance

We found no evidence to support the exclusion of female participants on statistical grounds. Individuals within groups were more different from each other than the groups were from each other. As a result, sex alone did not directly correlate with biological variance analyzed here. We demonstrate techniques for classifying individuals based on patterns in their physiology, and this approach could be adopted to identify new groups that have more in common than those grouped by older but less data-driven categories, like a binary “sex”.

## Conclusion

This work supports the use of sex as a biological variable in biomedical research, while not supporting the still-commonly held concern that including females as subjects increases variance and weakens analysis power. Not only do cyclic females not accumulate greater measurement error over time than do males, but the majority of unexplained variance is within sex category, not between them. There are no doubt situations—like breast cancer or pregnancy—where sex differences create large effects, but these differences cannot be used to relegate research on females to these special cases. Females still need to be more routinely included in research, and we find no statistical evidence that doing so would negatively affect study power. Physiological data-driven categorizations are likely to control for structured variance more precisely over time than are traditional demographic variables (i.e., sex, age, among others). For this reason, all subjects are worthy of inclusion in more time series analyses.

## Methods

### Data source and preprocessing

All data were part of the TemPredict Study [30]. This included physiological data generated using the wearable device Oura Ring (Oura Health Oy, Oulu, Finland), as well as survey data such as self-reported sex and age. Nightly aggregated and high-resolution (per minute or per 5 min) data were provided and stored in large parquet files on the San Diego supercomputer (SDSC) and accessed via the Nautilus Portal [31].

For each participant, a single parquet file for nightly data, also referred to as sleep summary data, contains sleep-related data fields (sleep time start, sleep time end) and the aggregated data fields: temperature max, temperature trend deviation. A single row with the longest sleep duration value for each date was chosen to ensure a single set of measurements per night. High-resolution physiological data contain distal body temperature and metabolic activity metrics (MET) recorded at 1-min intervals 24 h per day. Preprocessing required the creation of date-time indexing, normalization of indexes to a ‘local-time’, removal of duplicate time points, filtering of values below the 0.5 quantile and above 0.95 quantile for each participant, and annotation of awake or asleep based on information contained in the nightly summary data. Temperature values for timepoints where corresponding MET recordings were lower than 0.5 were dropped to remove potential artifacts from the data caused when a user was not wearing the device, either when charging the device, as elevated temperatures are often recorded at the start of charging, or for other unknown reasons.

### Subjects

63,153 owners of an Oura Ring were identified as having suitable wearable data. From these, 62,653 also had associated survey responses to the question “What is your biological sex? Male, Female, Other (please describe).” From this data set, 39.9% identified as female and 83.4% as white (Table 2). To generate a cohort with little data missingness, participants were chosen only if all data type files were available and if temperature data were present for all months between January and November 2020 (*n* = 7915). Further filtering eliminated participants' whose temperature data showed less than 70% average daily completeness. From the filtered participant list, we generated a cohort of 600 self-reporting females and males, such that the ages of participants generated an even distribution across six age bins spanning from 20 to 80 years old, with 50 individuals per age bin.

**Table 2:** Demographics of the full cohort and 300 F/M cohort

Demographic variables	Full cohort	300 F/M cohort
*n*	62,653	600
Age, mean (SD)	44.3 (12.3)	49.4 (16.4)
Sex, *n* (%)		
Female	25,001 (39.9)	300 (50.0)
Male	37,597 (60.0)	300 (50.0)
Other	55 (0.1)	0 (0.0)
Race, *n* (%)		
African	98 (0.02)	1 (0.2)
Asian	3292 (5.3)	18 (3.1)
Black	882 (1.4)	6 (1.0)
Ethnic other	2232 (3.6)	27 (4.5)
Middle eastern	602 (1.0)	3 (0.5)
Multiple	1688 (2.7)	15 (2.5)
Native American	85 (0.1)	0 (0.0)
Native Hawaiian	126 (0.2)	1 (0.2)
South Asian	936 (1.5)	2 (0.3)
White	50,012 (79.8)	511 (85.16)
Missing	2700 (4.3)	16 (2.7)
Hispanic ethnicity, *n* (%)		
No	58,509 (93.4)	575 (95.8)
Yes	3709 (5.9)	19 (3.2)
Skipped	435 (0.7)	6 (1.0)
Age bin, *n* (%)		
18–19	245 (0.4)	0 (0.0)
20–29	6958 (11.1)	100 (16.7)
30–39	16,737 (26.7)	100 (16.7)
40–49	18,103 (28.9)	100 (16.7)
50–59	12,941 (20.7)	100 (16.7)
60–69	5857 (9.3)	100 (16.7)
70–79	1684 (2.7)	100 (16.7)
80+	128 (0.2)	0 (0.0)

### Analysis methods

#### Autocorrelation clustering

Autocorrelation is the correlation of a time series signal that is linearly related to a lagged version of itself and is often used to find repeating patterns such as periodic signals. In the case of continuous temperature monitoring, the nightly aggregated temperature trend deviation autocorrelation signal for cyclic individuals shows a wave-like pattern, whereas the same analyses for acyclic individuals do not. To automate classification of the signal as cyclic or acyclic, autocorrelation was calculated for each individual using 6 months of nightly summary data and pairwise distances of each signal was calculated with dynamic time warping. Hierarchical clustering was performed next to systematically separate participants based solely on the distance between the autocorrelation series. Autocorrelation was performed with the *acf* tool in the *statsmodels* (version 0.13.5, https://www.statsmodels.org/) python package [32]. Pairwise distances of each signal was calculated using the dynamic time warping tool *fastdtw* (version 0.3.4, https://pypi.org/project/fastdtw/) [33], and hierarchical clustering was performed using the *cluster.hierarchy.linkage* from the *scipy* [34] package (version 1.10.1, https://scipy.org/).

#### Wavelet analysis

Wavelet transform is a signal processing technique for detecting dominant modes of variability and the time dependence of those variations of power in time–frequency space [35]. Following identification of acyclic and strongly cyclic participants through clustering and identification of weakly cyclic individuals by manual inspection of autocorrelation plots, we performed wavelet transforms with sleep summary temperature maximum data, by a sampling of once per day to generate the power spectra (package *pywt* (version 0.4.0b0, https://pywavelets.readthedocs.io/) [36], Morlet mother wavelet). Average power for each participant was calculated for the 26- to 32-day band and average and standard error of each category was calculated and plotted for comparison.

#### Mean and variance of temperature by sex

Temperature mean and variance was calculated for each participant at three different time states (24 h, when awake, and when asleep) by sub-setting to each time state, generating an hourly rolling average, and then calculating either the average or overall variance. The average and standard deviation of either mean or variance was calculated for each category (cyclic female, acyclic female, acyclic male) and statistical significance between groups was calculated using the Mann–Whitney–Wilcoxon two-sided test with Bonferroni correction for 9 comparisons using the add_stat_annotation function from the *statannot* (version 0.2.3, https://pypi.org/project/statannotations/) python package.

#### Mean temperature by age bin

Using the individually calculated mean temperature described above, the mean and standard deviation of each age bin group was calculated and compared using a Mann–Whitney–Wilcoxon two-sided test with Bonferroni correction for 15 comparisons.

#### Cohen’s d

To measure the magnitude of the difference between the temperature mean and variance of the three categories (cyclic female, acyclic female, and acyclic male), we calculated the Cohen’s d effect size [37] using the *compute_effsize* function in the *pingouin* (version 0.5.3, https://pingouin-stats.org/) library [38].

#### Coefficient of variation (CV)

A common metric for assessing temporal variability, CV is a measurement of dispersion and determines the variability of measurements relative to the mean of the population, a ratio of the standard deviation to the mean; CV = standard deviation x mean^−1^ [39].

#### Proportional variability index (PV)

The proportional variability (PV) index, a metric developed to measure temporal variability without some of the shortcomings of CV, such as dependence on the mean of the measurements and sensitivity to rare events, quantifies variability as an average percent difference between all possible combinations of measurements in a time series [39–42]; PV = 2 [ ∑^z^(1 −  (min(*z*_*i*_, *z*_*j*_)/max(*z*_*i*_,*z*_*j*_))]/(*n*(*n* − 1)), where *n* = total number values, *z* = a list of values on which pairwise comparisons are calculated, *i* and *j* = indices of any two different values.

#### Consecutive disparity index (*D*)

The consecutive disparity index (*D*) determines the average rate of change between consecutive values in a time series [39] and accounts for the shortcomings of CV along with keeping the ordering of measurements in time; *D* = (1/(*n* − 1)) ∑_i-1_^*n*−1^ |ln (*p*_*i*_ + 1 / *p*_*i*_)|, *n* = length of time series, *p*_*i*_ = value in series at time *i*).

#### Cumulative error rates

As previously described [16], if random error equals a distance between a measurement and the expected value of the measurement, cumulative error represents the error accumulated over time when compared to the expected value, here defined as greater than one standard deviation (SD) from a comparative mean. This was designed to simulate the likelihood of an individual receiving only a single randomly timed measurement being more than 1 SD from the mean of the population to which they are being compared. For assessing cumulative static error for the three groups, cyclic females, acyclic females, and acyclic males, error was calculated over several time durations. For each individual, static error is calculated by subtracting the mean of the individual’s population (*m*_*p*_) from the temperature measurement at each time point (*t*_*i*_), then dividing by the mean standard deviation of the population (*s*_*p*_) (SE = ((*t*_*i*_ − m_p_)/*s*_*p*_) − 1). Individual cumulative error for each timepoint is simply the sum of all time points prior to the current measurement. Kruskal–Wallis test was performed with the *scipy* (version 1.10.1, https://scipy.org/) package *stats.kruskal* test to compare the final cumulative sums of the individuals within the three groups [34]. Bonferroni correction was applied to the threshold of significance by dividing 0.01 by 4 to account for the 4 different comparisons.

## Data Availability

Oura’s data use policy does not permit us to make the data available to third parties. Therefore, those seeking to reproduce findings in this manuscript should contact the corresponding author B.L.S. Distribution of the source code is limited by the Department of Defense and therefore it cannot be shared.
