# Correction: Variability of temperature measurements recorded by a wearable device by biological sex

**DOI:** 10.1186/s13293-023-00568-x

**Published:** 2023-11-13

**Authors:** Lauryn Keeler Bruce, Patrick Kasl, Severine Soltani, Varun K. Viswanath, Wendy Hartogensis, Stephan Dilchert, Frederick M. Hecht, Anoushka Chowdhary, Claudine Anglo, Leena Pandya, Subhasis Dasgupta, Ilkay Altintas, Amarnath Gupta, Ashley E. Mason, Benjamin L. Smarr

**Affiliations:** 1 Department of Biomedical Informatics, UC San Diego Health, University of California San Diego, San Diego, CA USA; 2 Gene Lay Department of Bioengineering, University of California San Diego, 9500 Gilman Dr, La Jolla, San Diego, CA USA; 3 Bioinformatics and Systems Biology, University of California San Diego, San Diego, CA USA; 4 Department of Electrical and Computer Engineering, University of California San Diego, La Jolla, CA USA; 5 Osher Center for Integrative Health, University of California San Francisco, San Francisco, CA USA; 6 Department of Management, Zicklin School of Business, Baruch College, The City University of New York, New York, NY USA; 7 Halıcıoğlu Data Science Institute, University of California San Diego, San Diego, CA USA; 8 San Diego Supercomputer Center, University of California San Diego, San Diego, CA USA

**Correction: Biology of Sex Differences (2023) 14:76** 10.1186/s13293-023-00558-z

Following publication of the original article [1], the authors reported an error in their equal contributions statement. The statement should read: Ashley E. Mason and Benjamin L. Smarr contributed equally and are co-senior authors. The original article [1] has been updated.
